# Supplementary material for: Splice-Junction-Based Mapping of Alternative Isoforms in the Human Proteome
Source: Cell Rep. Author manuscript; Available in PMC 2020 Jan 15. (PMC6961840; doi:10.1016/j.celrep.2019.11.026)
Supplement: 2 [file NIHMS1546469-supplement-2.zip › DF1/PXD006675/RightAtrium/RightAtrium_31_ELN_AQLLPGALAAAK.pdf]

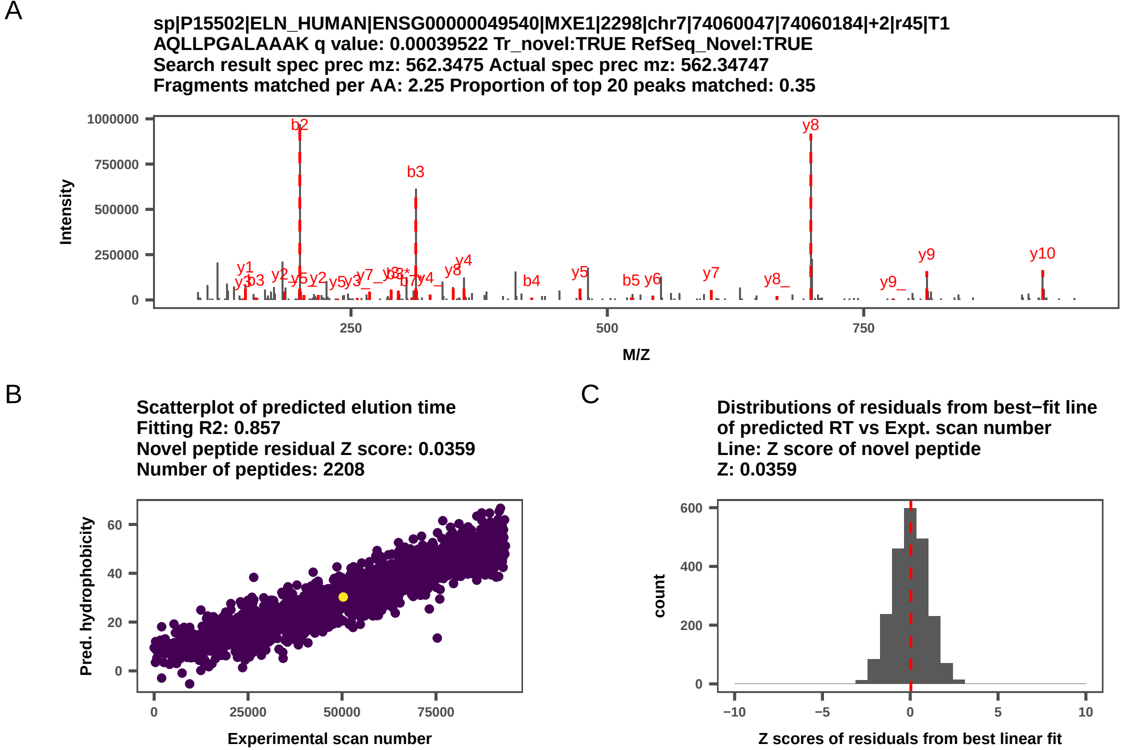
A
sp|P15502|ELN_HUMAN|ENSG00000049540|MXE1|2298|chr7|74060047|74060184|+2|r45|T1
AQLLPGALAAAK q value: 0.00039522 Tr_novel:TRUE RefSeq_Novel:TRUE
Search result spec prec mz: 562.3475 Actual spec prec mz: 562.34747
Fragments matched per AA: 2.25 Proportion of top 20 peaks matched: 0.35
Intensity
1000000
750000
500000
250000
0
b2
b3
y8
y9
y10
y4
b4
y5
b5
y6
y7
y8_
y9_
M/Z
250
500
750
B
Scatterplot of predicted elution time
Fitting R2: 0.857
Novel peptide residual Z score: 0.0359
Number of peptides: 2208
Pred. hydrophobicity
60
40
20
0
Experimental scan number
0
25000
50000
75000
C
Distributions of residuals from best−fit line
of predicted RT vs Expt. scan number
Line: Z score of novel peptide
Z: 0.0359
count
600
400
200
0
Z scores of residuals from best linear fit
−10
−5
0
5
10
